# Mitochondrial translation initiation machinery: Conservation and diversification^☆^

**DOI:** 10.1016/j.biochi.2013.07.024

**Published:** 2014-05

**Authors:** Anton Kuzmenko, Gemma C. Atkinson, Sergey Levitskii, Nikolay Zenkin, Tanel Tenson, Vasili Hauryliuk, Piotr Kamenski

**Affiliations:** a University of Tartu, Institute of Technology, Nooruse 1, Tartu, Estonia; b Molecular Biology Department, Faculty of Biology, M.V. Lomonosov Moscow State University, 1/12 Leninskie Gory, 119991 Moscow, Russia; c Centre for Bacterial Cell Biology, Institute for Cell and Molecular Biosciences, Newcastle University, Newcastle upon Tyne NE2 4AX, United Kingdom; d Department of Molecular Biology, Umeå University, Umeå, Sweden; e Laboratory for Molecular Infection Medicine Sweden (MIMS), Umeå University, Umeå, Sweden

**Keywords:** Mitochondria, Ribosome, IF2, IF3, Translational activators

## Abstract

The highly streamlined mitochondrial genome encodes almost exclusively a handful of transmembrane components of the respiratory chain complex. In order to ensure the correct assembly of the respiratory chain, the products of these genes must be produced in the correct stoichiometry and inserted into the membrane, posing a unique challenge to the mitochondrial translational system. In this review we describe the proteins orchestrating mitochondrial translation initiation: bacterial-like general initiation factors mIF2 and mIF3, as well as mitochondria-specific components – mRNA-specific translational activators and mRNA-nonspecific accessory initiation factors. We consider how the fast rate of evolution in these organelles has not only created a system that is divergent from that of its bacterial ancestors, but has led to a huge diversity in lineage specific mechanistic features of mitochondrial translation initiation among eukaryotes.

## Highlights

• Mitochondrially-encoded proteins are mostly respiratory chain components.
• The mitochondrial translation system is thus organized in a very specific way.
• Initiation involves mRNA-specific activators and bacteria-like initiation factors.
• We show that *Saccharomyces cerevisiae* Aim23p is a functional ortholog of bacterial IF3.
• We review the lineage specific features of mitochondrial translation initiation.

## 1 Introduction

The mitochondria of eukaryotic cells provide energy via the process of oxidative phosphorylation, perform fatty acid, heme and iron-sulfur cluster biosynthesis, and coordinate programmed cell death [1]. According to the generally accepted endosymbiotic theory, the ancestor of these organelles was a free-living bacterium that survived engulfment to become incorporated as an obligate endosymbiont within the cytoplasm of the host cell [2]. During the course of evolution, most of the mitochondrial protein-coding genes have been transferred to the nuclear genome. However, a few genes have been retained in the genome of the modern organelle. The gene complement can differ species to species, but mostly codes for ribosomal RNAs, tRNAs and membrane components of the electron transport chain. The mitochondrial genome encodes just 8 proteins in yeast [3], and 13 in humans [4]. The presence of a protein-coding genome, although small, necessitates the preservation of a functional translation apparatus in mitochondria.

The mitochondrial protein synthesis system has a similar architecture to that of its bacterial relatives, with the translational cycle subdivided into four universal steps: initiation, elongation, termination and recycling. Although there are many conserved aspects, mitochondrial translation is characterized by a number of distinctive features that set it apart from bacteria [5]. The mitochondrial ribosome is characterized by a higher protein content in comparison with the bacterial counterpart [6]. The mitochondrial genetic code deviates from the standard, with differences in codon usage accompanied by a reduction in number and modifications of mitochondrial tRNAs [7].

One of the most dramatic differences between mitochondrial and bacterial translation is in the translational factors orchestrating the process, especially initiation factors. In bacteria, there are three universally present initiation factors, IFs: IF1, IF2, and IF3 [8]. Mitochondrial IF2 (mIF2) is universally present, mIF3 is near-universal, with a handful of exceptions, and mIF1 is universally lacking [9]. Finally, there is a large group of lineage specific mitochondrial translational activators, the majority of which have been identified in the yeast *Saccharomyces cerevisiae* [9,10]. In this review we summarize the current knowledge about protein factors involved in mitochondrial initiation by contrasting it with the ancestral bacterial system and paying special attention to lineage specific features.

## 2 Mitochondrial initiation factor 2 (mIF2)

### 2.1 General characteristics of the bacterial ortholog

**Fig. 1:**
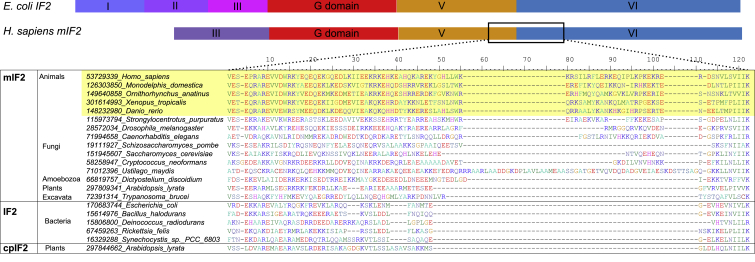
Domain organization of IF2 and mIF2. Location and sequence alignment of the mIF2 insertion region is shown for a set of representative species. The yellow highlighting shows the taxonomic limits of the conserved insertion region. See Ref. [9] for a larger alignment.

IF2 is a translational GTPase that orchestrates initiator tRNA selection and ribosomal subunit joining (for review see Ref. [11]). The latter activity is conserved among IF2 and its orthologs in the eukaryotic cytoplasmic translation system (eIF5B) and archaea (aIF5B) [12]. IF2 consists of six domains numbered from I to VI (Fig. 1). Domain IV is a GTPase, and domain VI directly interacts with the initiator Met-tRNA_i_^Met^ [13].

### 2.2 Functions of mIF2

The first function of mIF2 is selection of the initiator tRNA. Unlike in bacteria, in human mitochondria one methionine tRNA species acts both as initiator tRNA and elongator tRNA [14]. A fraction of the Met-tRNA^Met^ is formylated, leading to an increase in tRNA affinity to mIF2, accompanied with a decrease in affinity to EF-Tu – a translational GTPase delivering elongator tRNAs during the elongation stage. This ensures that formylated fMet-tRNA^Met^ specifically participates in the initiation of translation [15]. This dual use of Met-tRNA^Met^ is not limited to mammals; the single celled excavate parasite *Trypanosoma brucei*, which imports all its mitochondrial tRNAs, also formylates just a subset of Met-tRNA^Met^ molecules for use in initiation [16].

In yeast mitochondria, the situation is more similar to the bacterial system in that there are two tRNA^Met^ species, initiator (tRNA_i_^fMet^) and elongator (tRNA^Met^) [17]. As in the mammalian system, formylation of Met-tRNA_i_^fMet^ in *S. cerevisiae* increases its affinity to mIF2 [18]. In *Escherichia coli*, disruption of the *fmt* gene coding for Met-tRNA_i_^fMet^ formyltransferase abolishes initiator tRNA formylation, severely impairing bacterial growth [19], whereas in *Pseudomonas aeruginosa* the growth effect is only moderate [20]. A deletion of the equivalent gene *FMT1* in *S. cerevisiae* does not lead to a significant impairment of mitochondrial translation and yeast growth [21]. Moreover, replacement of *S. cerevisiae* mIF2 with its bovine ortholog in the context of the *FMT1* deletion also does not result in any visible defects of mitochondrial translation [22], suggesting that the relative insensitivity to formylation of initiator tRNA is a general feature of mIF2.

The relative insensitivity of *S. cerevisiae* to *FMT1* deletion has been suggested to be due to the participation of an accessory protein Aep3p in the process of initiator tRNA selection in *S. cerevisiae* mitochondria [23]. Simultaneous disruption of both *FMT1* and *AEP3* genes leads to a synthetic respiratory defect – a phenotype even more severe than that seen in *fmt*-deficient *E. coli* [23]. In vitro experiments have shown that complex formation between Aep3p and mIF2 promotes the binding of Met-tRNA_i_^fMet^ – but not of fMet-tRNA_i_^fMet^ – to mIF2, thus promoting Met-tRNA_i_^fMet^ use in initiation. Moreover, the genome of apicomplexan *Toxoplasma gondii* does not encode the FMT gene, suggesting that in this organism initiation naturally uses an unformylated initiator tRNA [24].

The second activity of IF2 and e/aIF5B – their role in ribosomal subunit joining – has not yet been experimentally investigated for mIF2. This is due to an absence of a suitable sophisticated mitochondrial in vitro translational system. Given that subunit joining is a universally conserved function of both bacterial (IF2) as well as eukaryotic and archaeal (e/aIF5B) orthologs, it is most likely that mIF2 has this activity as well. However, since mitochondrial translation has numerous unique characteristics, this is far from certain without direct experimental validation.

### 2.3 Role of the vertebrate-specific insertion in mIF2-ribosome interactions

Our understanding of mIF2 interactions with the ribosome is mainly based on a series of biochemical investigations using mutant variants of the protein [25] and a low-resolution structural reconstruction of bovine mIF2 in complex with initiator fMet-tRNA_i_^fMet^ on the *E. coli* ribosome [26]. Despite the overall homology between IF2 and mIF2, there are several differences. First, mIF2 lacks the first two domains of the bacterial factor [9] (Fig. 1). Second, it has an N-terminal mitochondrial targeting sequence, which is probably cleaved off upon import, though this has never been proven experimentally. Finally, a short vertebrate-specific insertion between domains V and VI was suggested to have an IF1-like function [27]. Deletion of this region in bovine mIF2 decreased the factor's affinity to the ribosome [25]. *E. coli* complementation experiments have demonstrated that expression of plasmid-borne bovine mIF2, but not *E. coli* IF2 can support the viability of an *E. coli* strain lacking genomic copies of initiation factors IF2 and IF1 [27]. This result was interpreted in a model postulating that, despite a lack of homology to IF1 and twice smaller size [9], the insertion serves as a functional replacement of IF1. Subsequent structural studies demonstrated that the insertion shares the same binding pocket on the bacterial ribosome as IF1 [26], seemingly supporting the idea that it has evolved as an IF1 substitute.

A phylogenetic analysis has been carried out in order to resolve the order of events in IF1 loss and gain of the mIF2-specific insertion [9]. This showed that the insertion region is highly variable in sequence and length among eukaryotes, with the full-length insertion limited in conservation to vertebrates, while mIF1 is universally lacking. This suggests that loss of IF1 predates the acquisition of the insertion, and therefore, the functionality of IF1 is not necessary for mitochondrial translation, irrespective of the presence or absence of the insertion. Bacterial IF1, as well as its cytoplasmic eukaryotic ortholog eIF1A are essential genes [28,29], acting as fidelity factors involved in initiator tRNA and start codon selection during the initiation complex assembly [30,31]. Translation initiation in mitochondria occurs on only a handful of different mRNAs, and is aided by a number of mRNA-specific activators (see below), and therefore it is likely that mitochondrial ribosomes do not face the fidelity problems that require the participation of IF1. The insensitivity of start codon selection to mutation of the initiation codon from AUG to AUA in the case of COX2 [32] and COX3 [33] mRNA underscores the relative lack of fidelity in selection of the initiator *codon* – in contrast with high fidelity in selection of the *position* of the start codon in the mRNA.

## 3 Mitochondrial initiation factor 3 (mIF3)

### 3.1 General characteristics of the bacterial ortholog

Bacterial IF3 is a translation factor that acts at the interface between ribosomal recycling – splitting of the post-termination complex into subunits – and translation initiation. During recycling, IF3 prevents re-association of the ribosomal subunits transiently separated by Elongation Factor G (EF-G) and the Ribosome Recycling Factor (RRF) [34,35]. During translation initiation, IF3 is involved in tRNA and mRNA selection, specifically destabilizing aberrant complexes [36,37]. IF3 is universally present in bacteria and near-universally present in mitochondria (see below) [9]. In the eukaryotic cytoplasm, the function of IF3 is carried out by an apparently non-homologous multisubunit factor, eIF3 [38].

**Fig. 2:**
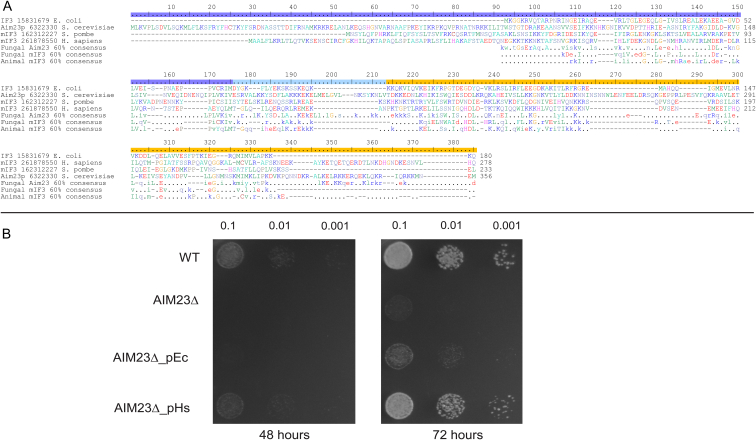
Human mIF3 rescues an *S. cerevisiae* strain lacking the genomic copy of AIM23, whereas *E. coli* IF3 has a weak, but detectable complementation activity. (A) mIF3/IF3 consensus sequences calculated at the 60% level using the Python script Consensus Finder [103]. See Ref. [9] for a larger alignment and three-dimensional location of conserved sites. Domain organization is indicated on the ruler above the alignment. (B) Restoration of mitochondrial functionality was assessed by growth of yeast strains on non-fermentable media YPGly requiring mitochondrial respiration. The genomic copy of AIM23 was knocked out with a gentamicin cassette resulting in AIM23Δ strain, which was complemented with plasmids expressing mIF3 from *S. cerevisiae* (WT), mIF3 from *H. sapiens* (AIM23Δ_pHs), or IF3 from *E. coli* fused with AIM23 mitochondrial import signal (AIM23Δ_pEc) under the control of *S. cerevisiae* 5′ and 3′ flanking regions. Yeast suspensions were spotted on the plate in ten-fold serial dilutions (OD_600_ is indicated above the spots) and incubated at 30 °C for 48 and 72 h.

Bacterial IF3 consists of globular N- and C-terminal domains connected with a flexible linker region [39] (Fig. 2A). In the bacterial 30S initiation complex, the C-terminal domain interacts with loop 790 of 16S rRNA, while the N-terminal domain can sample several conformations and interacts with the initiator fMet-tRNA_i_^fMet^ [13,40]. The protein is highly dynamic, both off [41] and on the ribosome [37], and formation of the 30S initiation complex with cognate tRNA drives IF3 into a conformation compatible with subsequent subunit joining [37]. Deletion experiments have shown that most of the factor's affinity to the ribosome resides in the C-domain, and N-terminally truncated factors are still functionally active [42].

### 3.2 Functions of mIF3

Similarly to its bacterial ortholog, mammalian mIF3 has been shown to promote both dissociation of the ribosome into subunits, and binding of the initiator tRNA to the ribosomal initiation complex [43]. As with bacterial IF3, the ribosome affinity of mIF3 is mostly dictated by the C-terminal domain, with a moderate contribution from the linker region [44]. mIF3 shares a proofreading function with IF3: it destabilizes initiation complexes that lack mRNA, or that are loaded incorrectly with elongator tRNAs, although the second activity is considerably weaker than in the case of IF3 [45,46]. Interactions of mammalian mIF3 with the ribosome have been mapped using chemical cross-linking followed by mass-spectrometry [47]. It was shown that mIF3 interacts with several ribosomal proteins that have bacterial homologues (MRPS5, MRPS9, MRPS10, MRPS18), as well as with some mitochondria-specific ribosomal proteins (MRPS29, MRPS32, MRPS36, PTCD3). Experiments with isolated N- and C-terminal domains of mIF3 have shown that only MRPS10 binds to the N-domain, while the rest of the ribosomal proteins interact with the C-domain.

In addition to these similarities, there are some specific features of mIF3. First, unlike in the case of IF3, addition of IF1 does not stimulate mIF3-dependent binding of initiator tRNA either to mitochondrial 55S or to bacterial 70S ribosomes [43]. However, since these experiments were performed in the presence of mammalian mIF2, one possible explanation is that the vertebrate-specific insertion in mIF2 interferes with IF1 binding, and therefore this effect does not reflect specific features of mIF3 *per se*. A similar experiment performed in the presence of bacterial IF2 and IF1, and mIF3 is required to resolve this question. Second mIF3 has N- and C-terminal extensions relative to bacterial IF3. Deletion of these regions leads, surprisingly, to an moderate increase in the factor's activity in a simplistic in vitro system, and a significant – ten-fold – increase in its affinity to the small subunit of the mitochondrial ribosome, 39S [45]. It was suggested that these extension regions safeguard against nonspecific associations with the small subunit. Deletions of mIF3 extensions do not change the profile of mIF3-ribosomal protein cross-linking, suggesting that these regions do not affect the topology of the factor's interaction with the 55S ribosome [47].

### 3.3 *S. cerevisiae* mIF3, Aim23p

All of the experimental results described above were obtained using either bovine or human mIF3 [43]. The *S*. *cerevisiae* ortholog, Aim23p, was not identified until a whole decade later [9]. Aim23p is highly divergent in sequence relative to mIF3, which precluded its early identification before the use of more sensitive sequence searching methods. As with mammalian mIF3 and bacterial IF3, Aim23p can be subdivided into N-terminal and C-terminal domains connected by a linker region. It also has several unique characteristics: an insertion in the linker region, and N- and C-terminal extensions that are longer than in the mammalian factor [9]. Phylogenetic analysis has shown that the distribution of *AIM23* is limited to Saccharomycetale yeast, similarly to that of the majority of mitochondrial translational activators identified to date (see below) [9].

Prior to its identification as the mIF3 orthologue, Aim23p had not been experimentally characterized, except for establishing that it is somehow important for mitochondrial functionality [48]. Subsequently, Aim23p's role as a *bona fide S*. *cerevisiae* mIF3 has been validated by complementation of a mitochondrial function deficiency caused by *AIM23* gene disruption in the presence of mIF3 from *Schizosaccharomyces pombe* [9]. Thus, despite the fact that mIF3 genes in *S. pombe*, *S*. *cerevisiae* and human are very divergent, particularly in comparison with *E. coli* IF3, these factors have conserved overlapping functions (Fig. 2A).

Since the human factor is the only mIF3 gene which is functionality proven in vitro, we have further validated Aim23p as mIF3 by performing similar complementation experiments using human mIF3 as well as *E. coli* IF3 fused to a mitochondrial localization signal (Fig. 2B, see Supplementary material for details). The human factor showed very strong complementation, almost to the wild type level, whereas *E. coli* IF3 had a weak, but detectable activity.

### 3.4 *S. cerevisiae*-specific proteins involved in translation initiation

In addition to mIF2 and mIF3, in *S*. *cerevisiae* three additional proteins were suggested to participate in recruitment of initiator tRNA: Aep3p, Rsm28p, and Rdm9p. Unlike translational activators (see below), these proteins do not seem to exert their functions via interactions with mRNAs, and act together with the ‘classical’ initiation factors.

Aep3p was first discovered as a protein stabilizing bicistronic ATP6/8 mRNA [49], and later the interaction of Aep3p with mIF2 was found to promote the recruitment of unformylated initiator tRNA [23] (see above). The second protein, Rsm28p, is associated with the small subunit of the mitochondrial ribosome and positively regulates translation of several mitochondrial mRNAs [50]. Moreover, expression of a mutated Rsm28p with an internal deletion of amino acids 120 to 186 suppresses growth defects caused by initiation codon mutations in *cox2* and *cox3* genes, indicating that this protein is likely to be involved in the selection of the initiation site [50]. Rsm28p physically and genetically interacts with mIF2 and the third protein, Rmd9p [51]. The exact function of this protein is not understood, although it has been hypothesized that it takes part in mRNA delivery to mitochondrial ribosomes [51].

## 4 Translational activators involved in mitochondrial translation initiation in *S. cerevisiae*

### 4.1 General characteristics

Translational activators are proteins that orchestrate mitochondrial translation in mRNA-specific ways [52]. They are involved in translation initiation, tethering of translating ribosomes to the membrane, and directing assembly of newly synthesized proteins into multiprotein complexes (Table 1).

The roles of individual activators in translation and post-translational incorporation of the polypeptides into complexes are often mutually exclusive, resulting in negative feedback control [10]: the activator promotes translation, then once the polypeptide is synthesized, the activator is sequestered by the completed protein, resulting in inhibition of its translation promotion activity. The activator can be released only upon the incorporation of the newly synthesized protein into its macromolecular complex. This complex is usually the respiration machinery or, in case of Var1p, the mitochondrial ribosome. This control loop ensures the correct stoichiometry of protein production in mitochondria.

The majority of *S. cerevisiae* activators are Saccharomycetes-specific in detectable homology [9]. Deletions of the majority of *S. cerevisiae* genes encoding translational activators leads to a complete loss of mitochondrial functionality, accompanied by a significant increase in life span [53]. For a detailed review of activator roles in processes downstream from initiation – protein assembly and ribosomal tethering – see Ref. [10].

A correlation between the abundance of translational activators in Saccharomycetes and the presence of long 5′ and 3′ untranslated regions (UTRs) in mitochondrial mRNAs of these organisms has been suggested [10,54], supported by experiments demonstrating direct interactions between activators and UTRs [55–57]. Mitochondrial mRNAs in fission yeast, *S. pombe*, lack 3′ UTRs, while 5′ UTRs are relatively short [58]. In mammals both 5′ and 3′ UTRs are virtually missing [59] and 5′ regions are generally devoid of secondary structures [60], mirroring considerably lower numbers of translational activators identified in these organisms so far. However, since a few mRNA-specific mitochondrial translational activators have been identified in plants [61] and humans [62] it may be that the lower number of these factors identified in other organisms is simply due to technical challenges. Moreover, the absence of long UTRs encoded in the mitochondrial genome does not necessarily translate into the absence of 3′ and 5′ extensions of mature mRNAs in every eukaryote: extensive mRNA editing in trypanosomal mitochondria regulates the efficiency of translation post-transcriptionally by altering the length and nucleotide composition 3′ the mRNA tails [63].

The mitochondrial genome of *S. cerevisiae* codes for eight proteins [3]. Seven of them (cytochrome b, cytochrome oxidases 1, 2, and 3, ATPase subunits 6, 8, and 9) are highly hydrophobic subunits of the mitochondrial respiration complexes integrated into the inner membrane, and the eighth (Var1p) is a protein of the small ribosomal subunit [64]. These eight proteins are translated from seven mRNAs; the open reading frame coding for Atp6p and Atp8p is bicistronic. The translational activators involved in translation of each of these mRNAs are described below.

### 4.2 Var1p

The translational activator of Var1p has recently been identified as Sov1p [53], and it was proposed that it interacts with, and stabilizes the 5′ UTR of VAR1 mRNA [10].

### 4.3 Cytochrome b (COB)

Five translational activators of COB have been discovered in *S. cerevisiae*: Cbs1p, Cbs2p, Cbp1p, Cbp3p and Cbp6p. The first two, Cbs1p and Cbs2p, interact with the 5′ UTR of COB mRNA [55,56], and co-purify only with mitochondrial ribosomes translating these mRNAs [65,66]. No interaction of Cbs1p and Cbs2p with naked ribosomes has been detected [65,66], suggesting that these translation activators are bound to COB mRNA during translation, rather than interacting with the ribosome directly. Similarly, Cbp1p also binds the 5′ UTR of COB mRNA [67]. This activator has a dual role; its interaction with mRNA is required both for its stabilization and translation [68,69]. The trinucleotide CCG in the 5′ UTR of COB mRNA was shown to be critical for Cbp1p binding [57]. As with Cbs1p and Cbs2p, no interactions with naked ribosomes for Cbp1p have been detected [69].

The two remaining translational activators of COB mRNA, Cbp3p and Cbp6p, do not seem to interact with the 5′ UTR of COB mRNA and are not involved in translation initiation *per se*. Instead, the Cbp3p·Cbp6p complex interacts with the ribosomal exit tunnel [70]. This interaction is absolutely required for synthesis of cytochrome b. The Cbp3p·Cbp6p complex also interacts with newly synthesized cytochrome ·, coordinating its synthesis with the assembly of *bc*_*1*_ complex of the respiratory chain [71].

Unlike the other translational activators of COB, the Cbp3p·Cbp6p complex is not a Saccharomycetes-specific feature of mitochondrial translation. Both proteins have homologues in *S. pombe* where they take part in the post-translational steps of cytochrome c reductase biogenesis [72]. Human homologues of these two proteins have also been found, though their functions have not been verified [10].

### 4.4 Cytochrome c oxidase subunit 1

The translation of Cox1p is regulated by two proteins, Pet309p and Mss51p. Pet309p is a member of the pentatricopeptide repeat (PPR) protein family – a large set of proteins with members participating in RNA editing, RNA splicing, RNA cleavage and translation in mitochondria and chloroplasts [73]. Pet309p is anchored in the mitochondrial inner membrane [74], and its interaction with the COX1 mRNA 5′ UTR is necessary for COX1 translation [75]. In addition to its role in translation, Pet309p also promotes the stability of un-spliced COX1 pre-mRNA. Pet309p specifically stabilizes the intron-containing version of COX1 mRNA while having no effect on the stability of mature mRNA [75].

Mss51p regulates the level of Cox1p expression by acting simultaneously as a positive and negative effector: interactions of Mss51p with the 5′ UTR and the coding region of COX1 promote its translation [76,77], whereas interactions with newly synthesized Cox1p have an inhibitory activity on translation [76]. This dual mode of action mediates the correct assembly of the respiratory complex. The *S. pombe* Mss51p homologue does not activate translation of Cox1, sharing only the post-translational inhibitory activity with *S. cerevisiae* [72].

### 4.5 Cytochrome c oxidase subunit 2

Translation of COX2 mRNA is regulated by a single activator, Pet111p, via a direct interaction with a stem-loop structure in the COX2 5′ UTR [78–80]. An excess of Pet111p is associated with an increase in Cox2p synthesis [81] accompanied by inhibition of Cox1p synthesis [82], most likely via unproductive interactions with factors involved in Cox1p synthesis.

### 4.6 Cytochrome c oxidase subunit 3

Synthesis of Cox3p is regulated by three translational activators: Pet54p, Pet122p and Pet494p, which all bind the 5-UTRs of the COX3 mRNA 480 to 330 nucleotides upstream of the start codon [83–87]. Most of the Cox1p, Cox2p and Cox3p translational activators (namely Pet309p, Pet111p, Pet54p, Pet122p, and Pet494p) also interact with each other and form a large complex associated with the matrix surface of the inner mitochondrial membrane, ensuring that all three mitochondrially-encoded subunits are co-synthesized in physical proximity to one another [88].

### 4.7 ATPase subunits 6/8 and 9

Two of the three mitochondrially-encoded yeast ATPase subunits, Atp6p and Atp8p, are synthesized from one bicistronic transcript, translation of which is regulated by a single translational activator, Atp22p [89]. The synthesis of Atp6p and Atp8p depends on that of F_1_ ATPase subunit, defects of which can be complemented by overexpression of Atp22p [90].

Two proteins have been found to be specifically required for Atp9p synthesis: Aep1p (or Nca1p) [91] and Aep2p (or Atp13p) [92–94]. However, no binding of Aep1p to either the ATP9 mRNA or yeast mitochondrial ribosomes has been detected, even though the suppression of mutations in this protein by a point mutation in the 5′ UTR of ATP9 mRNA [95] suggests the existence of a direct interaction.

## 5 Conclusions and outlook

The differences in the molecular machinery of mitochondrial and bacterial translational systems reflect, at least partially, their respective adaptations to the very different decoding challenges they meet. Bacterial ribosomes translate a wide variety of mRNAs, and selection of initiator fMet-tRNA_i_^fMet^ and the start codon is performed by the concerted action of three factors: IF1, IF2 and IF3. Mitochondrial ribosomes translate only a handful of mRNAs, but the products of these genes must be produced in the correct stoichiometry in order to ensure the correct assembly of the respiratory chain complex. Start codon selection by the ‘classical’ set of initiation factors is assisted by translational activators that position the ribosome on 5′ UTRs of transcripts, coordinating translation and incorporation of the complete protein into macromolecular complexes. Moreover, a specialized factor Aep3p is involved in initiator tRNA selection in *S. cerevisiae*. It may be that these ‘helper’ proteins are responsible for the ability of the mitochondrial system to make do without a universally conserved bacterial factor IF1. An alternative explanation that an insertion in mIF2 serves as mIF1 [27] is unlikely since the insertion is vertebrate-specific whereas mIF1 loss is universal in eukaryotes [9].

The divergence of mitochondrial translation initiation relative to that of bacteria may be a result of neutral drift fuelled by the high mutation rate of the mitochondrial genome [96]. Evolutionary drift can lead to an increase in complexity via fixation of mildly deleterious mutations that create a dependence on a new component in an interaction network of macromolecules [97]. The mitochondrial translational system, especially in yeast, relies heavily on numerous additional – in comparison to the bacterial system – accessory factors, the loss of which often leads to mitochondrial dysfunction (see Table 1). At the same time the ‘classical’ set of initiation factors is highly divergent: mIF2 is missing two N-terminal domains [9] which in its bacterial counterpart are involved in the interactions with the ribosome [98], and mIF3 has considerably weakened tRNA proofreading activity in comparison with IF3 [45]. While increasing the *total* complexity of the system, this evolutionary ratchet has lead to simplification of some of its *aspects*: in the presence of numerous accessory factors, the mitochondrial system is no longer dependent on mIF1, leading to its loss. Relaxed selection has led to an accumulation of extension and insertion segments in mIFs – a feature also characteristic of mitochondrial ribosomal proteins [99] and mitochondrial Elongation Factor Tu (EF-Tu) in some lineages [100] – and we argue that in the case of vertebrate mIF2, opportunistic expansion of one such a segment occupying the IF1-binding pocket has increased the factor's affinity to the ribosome and led to insensitivity of the mitochondrial system to bacterial IF1 [43].

Our understanding of translation initiation in mitochondria is far from complete. Recent identification of *S. cerevisiae* Aim23p as the mIF3 ortholog [9] paves the way for in vivo experimentation with this factor in a highly genetically amenable organism. The role of mIF2 in subunit joining – the core function shared between bacterial IF2 and its eukaryotic orthologue, eIF5B – still remains to be tested experimentally. Lastly, the recent application of the ribosome profiling technique [101] to analysis of organellar translation [102] promises to revolutionize investigations of regulation of mitochondrial translation.

## Figures and Tables

**Table 1:** Yeast translational activators and mRNA-nonspecific accessory factors involved in translational initiation.

Target mRNA	Activator	Respiratory growth of *S. cerevisiae* deletant/mutant strain	Interacts with/Functional role	Orthologs outside Saccharomycetes [9,10]
*Translational activators*				
VAR1	Sov1	No [53]	No experimental data	No
COB	Cbs1	No [104]	5′ UTR [55] Mitochondrial ribosomes [66]	No
	Cbs2	No [104]	5′ UTR [55] Mitochondrial ribosomes [66]	No
	Cbp1	No [105]	5′ UTR [67]	Yes (only in other fungi)
	Cbp3·Cbp6	No [106]	Mitochondrial ribosomes [70]	Yes

COX1	Pet309	No [75]	5′ UTR [75]	No
	Mss51	No [107]	5′ UTR [77], mRNA coding part [77], Cox1 protein [76]	Yes (only in other fungi)

COX 2	Pet111	No [78]	5′ UTR [78]	No
COX 3	Pet54	No [85]	5′ UTR [85]	No
	Pet122	No [85]	5′ UTR [85]	No
	Pet494	No [85]	5′ UTR [85]	No

ATP6/8	Atp22	No [89]	5′ UTR [89]	No
ATP9	Aep1	No [91]	Possibly 5′ UTR [95]	No
	Aep2	No [92]	No experimental data	Yes

*mRNA-nonspecific accessory factors involved in translational initiation*				
	Aep3	No [49]	Stabilizes bicistronic ATP6/8 mitochondrial mRNA. Binds to mIF2 and supports the use of unformylated Met-tRNA_i_^fMet^ in initiation.	No
	Rsm28	Yes [50]	Mitochondrial ribosomal protein of the small subunit; genetic interactions suggest a possible role in promoting translation initiation.	No
	Rmd9	Slow growth [51]	Mitochondrial protein with role in delivering mRNAs to ribosomes; located on matrix face of the inner membrane and loosely associated with mitochondrial ribosomes.	No
