# Supplementary material for: Mitochondrial translation initiation machinery: Conservation and diversification
Source: Biochimie. 2014 May;100(100):132–40. doi: 10.1016/j.biochi.2013.07.024 (PMC3978653; doi:10.1016/j.biochi.2013.07.024)
Supplement: Supplementary file 1 [file mmc1.doc]

**Supplementary materials and methods**

**Plasmids:** The plasmid containing the AIM23 gene was described in . The plasmids expressing human mIF3 and *E. coli* IF3 were obtained by replacing the AIM23 ORF with the corresponding gene. For a detailed description of the procedure, see .

**Yeast strains:** The heterozygous AIM23 knockout diploid strain Y21294 carrying the chromosomal AIM23 gene disrupted by a geneticin (G418) resistance cassette was purchased from EUROSCARF. This strain was used to create two haploid strains with the disruption of the AIM23 genomic copy complemented by a plasmid expressing human mIF3 gene (AIM23∆_pHs), or the *E. coli* IF3 gene fused with the Aim23 mitochondrial import signal, amino acids 1-33 of *S. cerevisiae* Aim23p (AIM23∆_pEc). Two other haploid strains used in this work (lacking a saver plasmid, AIM23∆, and transformed with a plasmid expressing yeast AIM23, WT) were described in .

**In vivo complementation experiments:** The heterozygous diploid strain Y21294 was transformed by one out of two plasmids (either with the human mIF3 gene or with the *E. coli* IF3 gene fused with the Aim23 mitochondrial import signal) by electroporation . Positive clones were selected on a uracil-lacking medium using the plasmid URA3 gene as a marker. The resulting strains were subjected to sporulation followed by the dissection of resulting tetrads. Spores were selected by their ability to grow on the media with G418 (the corresponding resistance cassette is a marker of AIM23 gene disruption) and without uracil. As a result, two haploid stains without a functional AIM23 gene and either with the human mIF3 gene or with *E. coli* IF3 gene-containing plasmid were created.

Restoration of mitochondrial functionality was assessed by a respiration test. Ten-fold serial dilutions of the yeast strains (starting from OD_600_ 0.1) were spotted on a plate with glycerol-containing media, YPGly. Then the growth rates of these strains at 30°C were compared at different time points. As glycerol is a non-fermentable carbon source, this growth rate directly correlates with mitochondrial respiration.

**Bioinformatic analysis:** Full methods are described in. Briefly, sequences of mitochondrial translation factors were retrieved from the NCBI using the BlastP and PSI-Blast sequence searching algorithms, aligned with MAFFT and consensus sequences were generated for subsets of the alignment.

**Supplementary References:**

[1] G.C. Atkinson, A. Kuzmenko, P. Kamenski, M.Y. Vysokikh, V. Lakunina, S. Tankov, E. Smirnova, A. Soosaar, T. Tenson, V. Hauryliuk, Evolutionary and genetic analyses of mitochondrial translation initiation factors identify the missing mitochondrial IF3 in S. cerevisiae, Nucleic Acids Res 40 (2012) 6122-6134.

[2] J.R. Simon, K. McEntee, A rapid and efficient procedure for transformation of intact Saccharomyces cerevisiae by electroporation, Biochem Biophys Res Commun 164 (1989) 1157-1164.

[3] K. Katoh, K. Kuma, H. Toh, T. Miyata, MAFFT version 5: improvement in accuracy of multiple sequence alignment, Nucleic Acids Res 33 (2005) 511-518.
